# Impact of intracytoplasmic sperm injection in women with non-male factor infertility: A systematic review and meta-analysis

**DOI:** 10.3389/frph.2022.1029381

**Published:** 2022-10-28

**Authors:** Jun-Xia Huang, Yu-Qi Gao, Xiao-Tong Chen, Ying-Qi Han, Jing-Yan Song, Zhen-Gao Sun

**Affiliations:** ^1^ The First Clinical College, Shandong University of Traditional Chinese Medicine, Jinan, China; ^2^ School of Acupuncture, Moxibustion and Tuina, Shandong University of Traditional Chinese Medicine, Jinan, China; ^3^ Reproductive and Genetic Center of Integrated Medicine, The Affiliated Hospital of Shandong University of Traditional Chinese Medicine, Jinan, China

**Keywords:** intracytoplasmic sperm injection (ICSI), *in vitro* fertilization, non-male factor infertility, embryo laboratory outcomes, pregnancy outcomes, neonatal outcomes

## Abstract

**Objective:**

The purpose of this study is to determine whether intracytoplasmic sperm injection (ICSI) is beneficial in patients with non-male factor infertility.

**Methods:**

This systematic review and meta-analysis included articles from inception to May 2022. Published studies of non-male factor infertile women undergoing ICSI or *in vitro* fertilization (IVF) included in PubMed, Embase, web of science, Wanfang Database, and CNKI were searched by computer, without language restrictions. A random-effect model was applied to calculate the risk ratios (RRs) and their 95% confidence intervals (CIs). Letters, case reports, and review articles including meta-analyses and expert opinions were excluded. The primary endpoints were laboratory outcomes and pregnancy outcomes. The Secondary endpoints were neonatal outcomes.

**Results:**

Six randomized controlled studies and 20 retrospective cohort studies met the inclusion criteria. In meta-analytic forest plots, compared with IVF, those who received ICSI treatment were not different in fertilization rate (RR = 0.99, 95% CI [0.90–1.09], *P* = 0.88), total fertilization failure rate (RR = 1.30, 95% CI [1.17–1.45], *P* < 0.00001), and good quality embryo rate (RR = 0.94, 95% CI [ 0.86–1.02], *P* = 0.15), clinical pregnancy rate (RR = 0.84, 95% CI [0.70–1.01], *P* = 0.06), live birth rate (RR = 0.89, 95% CI [0.77–1.03], *P* = 0.13), miscarriage rate (RR = 1.06, 95% CI [0.78–1.43], *P* = 0.71), preterm neonatal delivery rate (RR = 0.92, 95% CI [0.67–1.26], *P* = 0.61), and low neonatal weight rate (RR = 1.13, 95% CI [0.80–1.61], *P* = 0.48). However, the implantation rate of IVF was better than ICSI (RR = 0.77, 95% CI [0.64–0.93], *P* = 0.005). In the subgroup analysis of the live birth rate of fresh embryo transfer, IVF performed in those ≥35 years had a higher live birth rate (RR = 0.82, 95% CI [0.78–0.83], *P* < 0.001).

**Conclusion:**

The findings of this study indicate that ICSI is not superior to IVF in the treatment of infertility related to non-male factors. In order to confirm this result, more high-quality clinical studies are needed.

## Introduction

Intracytoplasmic sperm injection is the first choice for male factor, and the sperm with the best viability and morphology are microscopically selected for injection into the oocyte, which greatly improves the fertilization rate and compensates for the shortcomings of conventional IVF. However, in recent years, the use of ICSI has far exceeded that of IVF in several countries, especially in non-male factors (1, 2). Many scholars believe that ICSI can improve the fertilization rate by avoiding the problems of fertilization failure that may result from traditional IVF method, and by reducing the cycle cancellation rate, so the clinical use of ICSI is advocated to be expanded. A clinical study comparing the impact of IVF and ICSI for non-man factor or unexplained infertility found an increased fertilization rate in patients using ICSI (3). However, the fertilization rate does not reflect the quality of embryos and pregnancy potential. The quality embryo rate reflects the quality of embryos, and implantation rate, clinical pregnancy rate, and live births reflect the developmental potential of embryos, so the outcome indicators to measure the impact of IVF and ICSI on non-male factor patients should be comprehensive. Some scholars have also extended the study to neonatal outcomes and assessed the advantage of ICSI in non-male factor patients by looking at the preterm birth rate, low birth weight rate, and malformation rate in neonates, although there was no significant difference, the safety of ICSI could be determined. To investigate the effect of ICSI versus IVF in non-male factor infertility patients, this study included 26 papers that met the requirements for meta-analysis to explore the differences between the two groups in embryonic laboratory outcomes and pregnancy outcomes, to provide more convincing evidence for clinical purposes.

## Materials and methods

### Inclusion criteria

(1) Study types were published in domestic and international literature with inclusion years from 2000 to 2022 examining the effects of ICSI and IVF on patients with non-male factor infertility, including randomized controlled studies and retrospective cohort studies.
(2) Study subjects: men with normal semen parameters; those with female factors associated with IVF indications (patients with infertility due to age or tubal factors, endometriosis, polycystic ovary syndrome, unknown causes, etc.).

### Exclusion criteria

(1) Study types of reviews, case reports, and conference reports were excluded; those with incomplete outcome indicators or unclear study conclusions were also excluded.
(2) Study subjects: men with severe oligospermia, weakness, and malformation; salvage ICSI studies; experimental animal studies; and those with PGT cycles.

### Group determination

The purpose of the study was to explore the application of ICSI and IVF among patients with non-male factors, divided into ICSI and IVF groups, to compare the differences between the ICSI and IVF groups in embryo laboratory outcome, pregnancy outcome, and neonatal outcome. As a result, we can prove whether ICSI is better than IVF.

### Search strategy

Computer searches of the major databases, PubMed, Embase, Web of Science, Wanfang Database, and CNKI, for studies that included all published articles in women with non-male factor infertility who underwent ICSI or IVF, were conducted according to the PICOS model: the study population was women with non-male factor infertility; the intervention population was patients with non-male factor infertility treated with IVF or ICSI; the comparison population was patients with non-male factor infertility treated with conventional IVF; the outcome indicators included primary outcome indicators (embryo laboratory outcome and pregnancy outcome) and secondary outcome indicators (neonatal outcome), and the types of included studies included randomized controlled studies and retrospective cohort studies. English literature was searched in PubMed as an advanced search with the search terms #1 male factor, #2 IVF/In vitro fertilization, and #3 ICSI/Intracytoplasmic sperm injection; the English search formula was (#1 AND #2 AND #3). The Chinese documents were searched in the WIFP and China Knowledge Network Journal Database, and the search form was #1 male factor, #2 IVF/In vitro fertilization, #3 ICSI/Intracytoplasmic sperm injection; the Chinese search form was (#1 AND #2 AND #3), and the search period is from the first publication of the journal to 2022.

### Data extraction and quality assessment

In the keyword search of several documents, the literature was screened according to the purpose of the study, information was obtained from the abstract, studies with little relevance were initially eliminated, secondary screening could be performed for unidentified literature, and two reviewers independently screened the literature and then cross-checked. Data were extracted from specific papers and systematically placed in tables to obtain the following study characteristics: study methodology (study design, study duration, sample size), study participants, and raw data on outcomes. The quality of the literature was evaluated with the Cochrane Risk of Bias Assessment Tool, scoring studies according to their actual situation in terms of sequence generation, participants, personnel, and allocation of outcome assessors concealed blinding, incomplete outcome data, selective outcome reporting, and other sources of bias.

### Outcome measures

The main observations were embryo laboratory outcomes including fertilization rate (defined as fertilized oocytes/total number of oocytes at MII stage ×100%), total fertilization failure rate (defined as cycles in which oocytes did not form 2 protoplasts/IVF cycles ×100%), good quality embryo rate (defined as D3 quality embryos or D5 quality blastocysts/number of cleaved embryos ×100%), implantation rate (defined as gestational sacs/number of embryos transferred ×100%) and pregnancy outcome, including fresh embryo clinical pregnancy rate, where clinical pregnancy is defined as intrauterine gestational sac and fetal heartbeat seen on vaginal ultrasound at least 4 weeks (defined as clinical pregnancy cycles/number of transfer cycles ×100%), miscarriage rate (defined as spontaneous miscarriage cycles within 28 weeks of gestation/number of clinical pregnancy cycles ×100%), and fresh embryo transfer live birth rate, where live birth was defined as delivery of a live fetus at gestational age ≥24 weeks,(defined as live births/number of embryo transfer cycles ×100%). Secondary observations for neonatal outcomes included neonatal preterm birth rate (number of cycles with gestational age less than 37 weeks/number of clinical pregnancy cycles ×100%), and neonatal low birth weight rate (defined as birth weight less than 2,500 g/total number of newborns ×100%).

### Statistical analysis

Statistical analysis was performed using RevMan 5.4 software. The two groups were analyzed comparatively and the effects of ICSI and IVF were evaluated based on the comparative results. The categorical data results were expressed as relative risk ratio RR and 95% confidence interval CI, the *I*^2^ statistical test was used to assess heterogeneity, and the *P* value and percentage of each result were reported. When *I*^2^ ≤ 50, there was little heterogeneity in the included studies and a fixed-effects model was adopted. When *I*^2 ^≥ 50%, the included studies were considered significantly heterogeneous and a random-effects model was adopted, followed by further subgroup analyses to explore the sources of heterogeneity. Sensitivity analysis was used to determine the stability of the results.

## Results

### Literature search results

**Figure 1:**
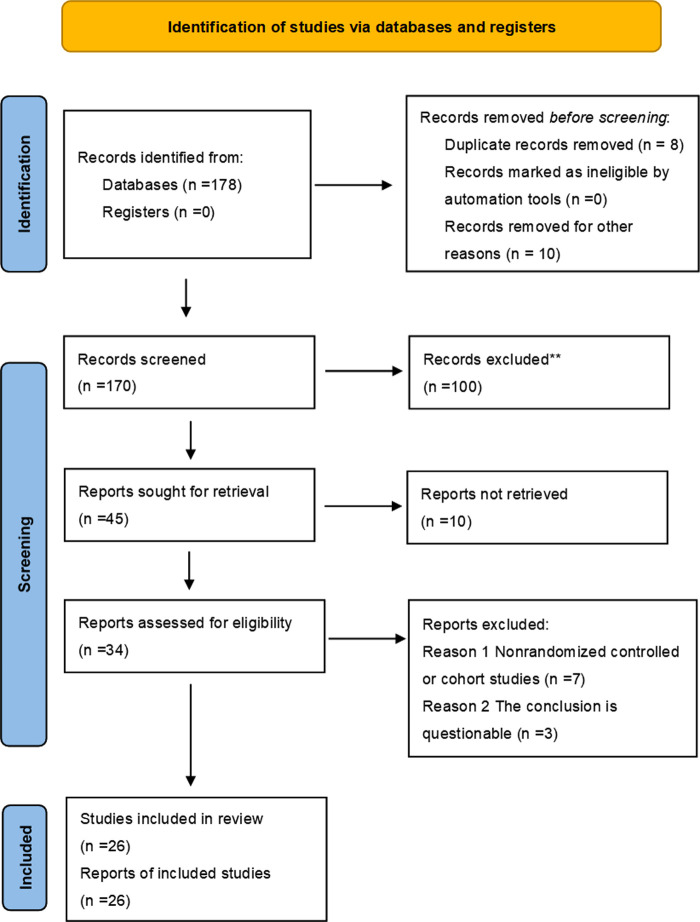
PRISMA checklist.

**Figure 2:**
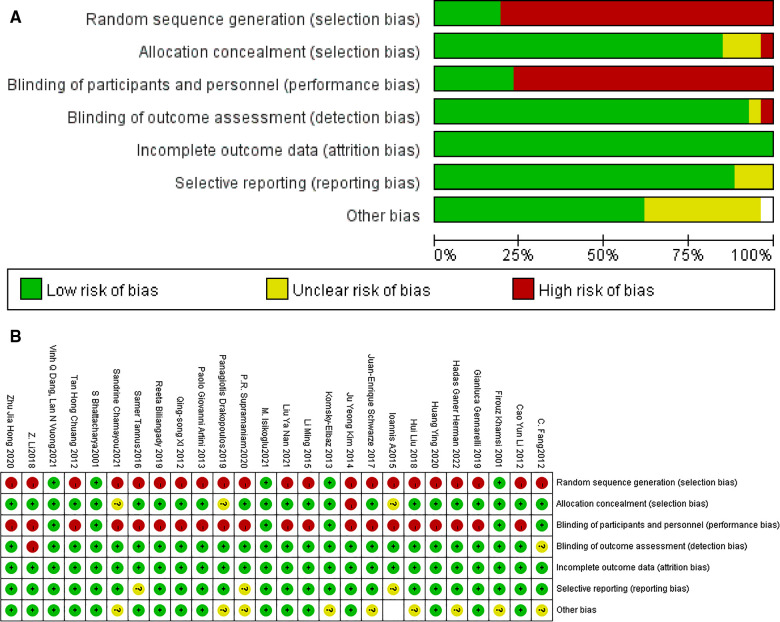
Low risk of bias, Unclear risk of bias, and High risk of bias were determined based on 1. whether there was random sequence generation 2. whether an allocation concealment scheme was implemented 3. whether blinding of patients, trial personnel, and outcome assessment was implemented 4. whether outcome data were complete 5. whether there was selective reporting 6. whether there was another bias.

A total of 178 articles were obtained during the initial review, including 96 articles in PubMed, and the types of articles were clinical trials and randomized controlled studies. 96 articles were retrieved and further screened according to “non-male factors”, and a total of 7 articles met the inclusion criteria, fourteen articles were included in the meta-analysis through similar articles and references involved in the study. 13 articles were retrieved from the China Knowledge Network journal database, 69 articles were retrieved from the Wan Fang Data Knowledge Service Platform, and 8 articles were duplicated. 5 Chinese articles meeting the requirements were included after screening, and 26 articles were finally included after comprehensive screening and evaluation according to the inclusion and exclusion criteria. As shown in Figure 1. The quality of the literature was evaluated using the Cochrane Risk of Bias Assessment Tool, as shown in Figure 2.

### Basic information of the included literature

Twenty-six eligible literature were finally included according to the purpose of the study (4–29) of which six were randomized controlled studies (6, 14, 17, 18, 25, 27) and 20 were retrospective cohort studies (4, 5, 7–13, 15, 16, 19–24, 26, 28, 29), Twenty-one English-language and five Chinese-language papers were included, and the literature was organized according to patient characteristics, patient numbers, study units, and primary outcome indicators. The basic characteristics of the collated literature are shown in Table 1.

**Table 1:** Basic characteristics of the included studies.

Study	Country	Period	Design	Fresh/frozen ET	Cycles (*n*)	ICSI group Age (years)	IVF group Age (years)	Inclusion criteria	Main outcome measures
						Mean ± SD	Mean ± SD		
Fang et al.	China	2007–2010	Retrospective cohort study	Fresh	80	36.64 ± 4.66	36.14 ± 4.57	GnRH-a long protocol; poor ovarian response	FR; CPR; good-quality embryo rate
Cao et al.	China	2008–2010	Retrospective cohort study	Fresh	403	34.85 ± 4.39	34.64 ± 4.76	25–46 years old; Cycles with only 1–3 oocytes collected	Cleavage rate; FR; complete fertilization failure rate
Khamsi et al.	Canada	–	Randomized controlled trial	Fresh	35	33.9 ± 5.46	33.9 ± 5.46	Age:21–44; GnRH-a long protocol	FR; good-quality embryo rate
Gennarelli et al.	Italy	2012–2018	Retrospective cohort study	Both	685	41 ± 0.8	41.1 ± 0.8	Age ≥40 years; Only autologous cycles; With unexplained infertility	FR; cumulative live birth rate; implantation rate
Herman et al.	Canada	2009–2017	Retrospective cohort study	Fresh	430	35.8 ± 4.3	35.2 ± 4.2	IVF with autologous oocytes	Obstetric outcome; placental histology
Huang et al.	China	2011–2018	Retrospective cohort study	Fresh	150	41.35 ± 1.54	41.72 ± 1.44	Patients of oocytes retrieved were less than 5	Normal fertilization rate; early abortion rate
Liu et al.	China	2011–2016	Retrospective cohort study	Fresh	486	41.32 ± 1.01	41.34 ± 1.08	40–43 years old; First ART cycle for IVF or ICSI	Clinical pregnancy; live birth; miscarriage rates
Ioannis A et al.	Britain	2009–2012	Retrospective cohort study	Fresh	151	41 ± 4.7	40.9 ± 3.7	Bologna criteria; With a single oocyte retrieved	LBR; FR; embryo formation rates
Schwarze et al.	Chile	2012–2014	Retrospective cohort study	Fresh	49,813	36.9 ± 4.4	36.3 ± 4.4	Patients of 155 ART clinics located in 15 Latin American	Fertilization failure; delivery rate; LBR
Kim et al.	Korea	2009–2013	Retrospective cohort study	Fresh	296	37.3 ± 4.9	33.9 ± 3.4	≤20% fertilization rate in a prior conventional insemination cycle	The total fertilization failure rate
Komsky-Elbaz et al.	Israel	2000–2011	Randomized controlled trial	Both	79	30.9 ± 3.5	30.9 ± 3.5	Patients with stages III and IV endometriosis;25–40 years	2PN FR; cleavage; blastocyst rate
Li et al.	China	2011–2013	Retrospective cohort study	Fresh	218	32.9 ± 4.1	32.9 ± 4.1	Age from 22 to 44 years; No indication of ICSI	Oocyte immaturity rate; available embryos
Liu et al.	China	2017–2019	Retrospective cohort study	Fresh	606	38.28 ± 2.35	37.97 ± 2.11	>35 years; <5 oocytes or >15 oocytes were recovered	FR; blastocyst formation rate; cleavage rate
M. Isikoglu et al.	Turkey	2015–2017	Randomized controlled trial	Fresh	138	30.45 ± 4.92	30.45 ± 4.92	Age <42 years; No TFF	FR; CPR; implantation and miscarriage rate
P.R. Supramaniam et al.	Britain	1991–2016	Randomized controlled trial	Fresh	1,376,454	18–50	18–50	Poor ovarian response (POR) cycles	Live birth (LB) per treatment cycle
Panagiotis et al.	Belgium	2009–2014	Retrospective cohort study	Both	4,891	34–38	34–38	Either IVF or ICSI for non-male factor infertility	2PN FR; embryo utilization rate; fresh LBR
Artini et al.	Italy	2007–2012	Retrospective cohort study	Fresh	425	38.23 ± 3.82	38.23 ± 3.82	Only one or two oocytes were retrieved	FR; cleavage rate; Implantation rate
Xi et al.	China	2009–2010	Retrospective cohort study	Fresh	406	36.1 ± 5.5	34.5 ± 4.6	More than three oocytes	FR; total fertilization failure; CPR
Biliangady et al.	India	2012–2017	Retrospective cohort study	Fresh	350	31.96 ± 4.1	30.71 ± 3.45	Age 25–35 years; normal ovarian reserve	FR; blastocyst formation rates; CRP; LBR
Tannus et al.	Canada	2012–2015	Retrospective cohort study	Fresh	745	41.2 ± 0.9	41.1 ± 0.9	Aged 40–43 years; underwent IVF treatments	LBR; FR; fertilization failure and embryo quality
Chamayou et al.	Italy	2011–2020	Retrospective cohort study	Fresh	211	33.9	33.9	Couples in which embryo culture was continued until day 5 were selected	Blastocyst rate
S Bhattacharya et al.	Britain	–	Randomized controlled trial	Fresh	435	31.6 ± 3.2	30.9 ± 4.1	<37 years; minimum acceptable semen characteristics	Implantation rate; pregnancy and fertilization rate
Tan et al.	China	2009–2011	Retrospective cohort study	Fresh	220	35.9 ± 5.1	34.7 ± 4.9	Patients with ≤5 oocytes were retrieved	FR; normal fertilization rate; cleavage rate
Dang et al.	Vietnam	2018–2019	Randomized controlled trial	Both	1,064	32.7 ± 4.6	32.7 ± 4.6	Undergone two or fewer previous IVF or ICSI	LBR
Li et al.	Australia	2009–2014	Retrospective cohort study	Both	21,072	34.6 ± 4.9	35.1 ± 4.6	At least one oocyte by either IVF or ICSI	Cumulative LBR
Zhu et al.	China	2016–2018	Retrospective cohort study	Both	659	41.9 ± 2.2	42.1 ± 2.6	Patients received mild stimulation for ovulation induction; female age ≥38 years	Cycle cancellation rate; pregnancy outcomes

ICSI, intracytoplasmic sperm injection; IVF, in vitro fertilization; ART, assisted reproductive technology; TFF, total fertilization failure; FR, fertilization rate; LBR, live birth rate; CPR, clinical pregnancy rate; GnRH-a long protocol, gonadotropin-releasing hormone (GnRH) agonist long protocols.

### Main outcome measures of effectiveness

#### Meta-analysis of embryo laboratory outcomes

##### The fertilization rates

**Figure 3:**
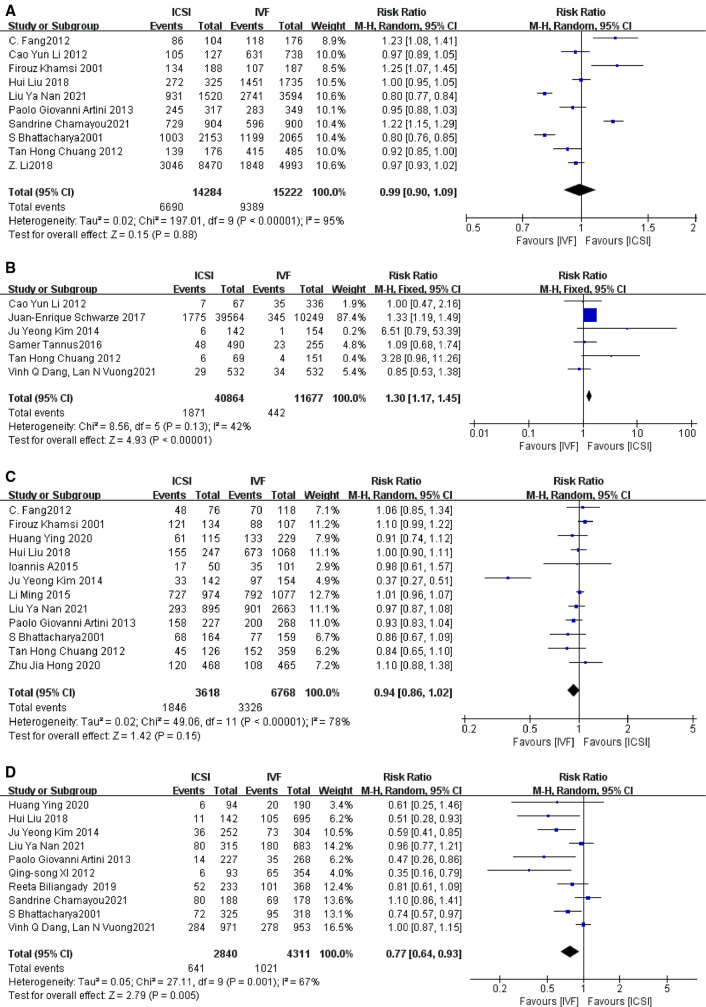
(**A**) Fertilization rate; (**B**) total fertilization failure rate; (**C**) good-good quality embryo rate; (**D**) implantation rate.

papers with fertilization rate outcome indicators supported by primary data were included according to the primary outcome indicators (3–6, 10, 16, 20, 24, 26, 28), and *I*^2^ was used to detect heterogeneity, and the relative risk RR and 95% CI were calculated by including the primary data. The *I*^2^ = 95%, *I*^2 ^> 50%, indicating a large heterogeneity among studies, so a random-effects model was adopted, and the results of the meta-analysis showed that the fertilization rate of the ICSI group was not statistically significant compared with the IVF group (RR = 0.90, 95% CI [0.90–1.09], *P* = 0.88, Figure 3A). Sensitivity analysis was performed for each study, and the two papers with the greatest weight were removed (16, 28), and the heterogeneity and *P*-values remained little changed across studies.

##### Subgroup analysis of fertilization rate

To determine the source of heterogeneity subgroup analysis was performed for the ICSI and IVF groups according to mean age, with mean age ≤35 years as a group and >35 years as a group. As shown in the Supplementary Figure S1, in the study of women with mean age <35 years, *I*^2 ^= 95% > 50%, there was large heterogeneity between studies and RR = 1.00, 95% CI was [0.89, 1.13], *P* = 0.97 > *P* = 0.05, the *P* value was not statistically significant, indicating that in women with younger mean age fertilization rate was not significantly different in these two groups. In the study of women with mean age greater than 35 years, *I*^2 ^= 98%, RR = 1.05, 95% CI [0.79, 1.40], *P* = 0.72 > *P* = 0.05, indicating that in the older group, fertilization rates in the ICSI group were not statistically significant compared to the IVF group, and the IVF group did not have better fertilization rates than the ICSI group.

##### The total fertilization failure rate

A total of six papers with primary data supporting indicators of total fertilization failure rate were included according to the main outcome indicators (5, 12, 13, 23, 26, 27), *I*^2^ = 42%, *I*^2^ < 50%, indicating close to moderate heterogeneity among studies, and a fixed-effects model was adopted, and the results of the meta-analysis showed that the total fertilization in the ICSI and IVF groups The results of the meta-analysis showed that the total fertilization failure rate was statistically significant in the ICSI group compared with the IVF group (RR = 1.30, 95% CI 1.17–1.45, *P* < 0.00001, Figure 3B), and it was concluded that the total fertilization failure rate was lower in the ICSI group than in the IVF group in the treatment of patients with non-male factor infertility by both assisted reproductive technologies. When sensitivity analysis was performed, it was found that after excluding the study with the greatest weight (12), RR = 1.11, 95% CI [0.83–1.48], *P* = 0.48 (Supplementary Figure S2), indicating that ICSI and IVF were not statistically significant in terms of total fertilization failure rate.

##### Subgroup analysis of total fertilization failure rate

Subgroup analysis of the ICSI and IVF groups according to patients from different countries was divided into foreigner and Chinese groups, as shown in the Supplementary Figure S3, in the subgroup of Chinese non-male factor infertility patients, *I*^2^ = 61%, RR = 1.64, 95% CI [0.52–5.15], *P* = 0.40. *I*^2^ in the subgroup of foreign non-male factor infertility patients was moderately heterogeneous, RR = 1.18, 95% CI [0.87–1.61], *P* = 0.29 indicating that even according to different countries of non-male factor patients for subgroup analysis, the total fertilization failure rates for ICSI and IVF were similar, and the total fertilization failure rate for ICSI were not superior to that of IVF.

##### Good quality embryo rate

A total of 12 papers with good quality embryo rate indicators supported by primary data (4, 6, 9–11, 13, 15, 16, 20, 25, 26, 29) were included according to the primary outcome indicators, with an *I*^2^ = 78%, indicating a large heterogeneity among studies. After excluding the study with the greatest weight (15), the *I*^2^ and *P* values did not vary significantly, indicating that the source of heterogeneity may be due to multiple factors. meta-analysis showed that the comparison of good quality embryo rate between ICSI and IVF was not statistically significant (RR = 0.94, 95% CI [0.86–1.02], *P* = 0.15, Figure 3C).

##### Subgroup analysis of good quality embryo rate

Because of the large heterogeneity, subgroup analysis was performed according to whether the patients had normal ovarian reserve function, with the number of oocytes ≤5 as a group and the number of normal oocytes obtained as a group. As shown in the Supplementary Figure S4 in the group of oocytes obtained ≤5, the *I*^2^ = 84%, with large heterogeneity among the 7 studies, and the relative risk RR = 0.85, 95% CI [0.71, 1.02], *P* = 0.08 > *P* = 0.05, indicating that there was no difference between the ICSI and IVF groups in terms of good quality embryo rate in the group with less than or equal to 5 eggs gained; in the normal egg gain group, the *I*^2^ = 31%, indicating less heterogeneity between studies, and the RR = 1.02, 95% CI [0.96, 1.08], *P* = 0.56 > *P* = 0.05, also indicating that there was no difference between the ICSI and IVF groups in terms of good quality embryo rate in the normal egg gain group. There was no difference in the rate of quality embryos between ICSI and IVF in the normal egg acquisition group.

##### Implantation rate

A total of 10 papers with implantation rate indicators supported by primary data were included according to the main outcome indicators (9, 10, 13, 16, 20–22, 24, 25, 27), with an *I*^2^ value of 67%, i.e., *I*^2^ > 50%, indicating a large heterogeneity among studies so a random effects model was adopted. When the study with the largest weight was removed (27), the results were no different. The results of the meta-analysis showed a statistically significant implantation rate in the ICSI group compared with the IVF group. (RR = 0.77, 95% CI [0.64–0.93], *P* = 0.005, Figure 3D).

#### Meta-analysis of pregnancy outcomes

##### Clinical pregnancy rate

**Figure 4:**
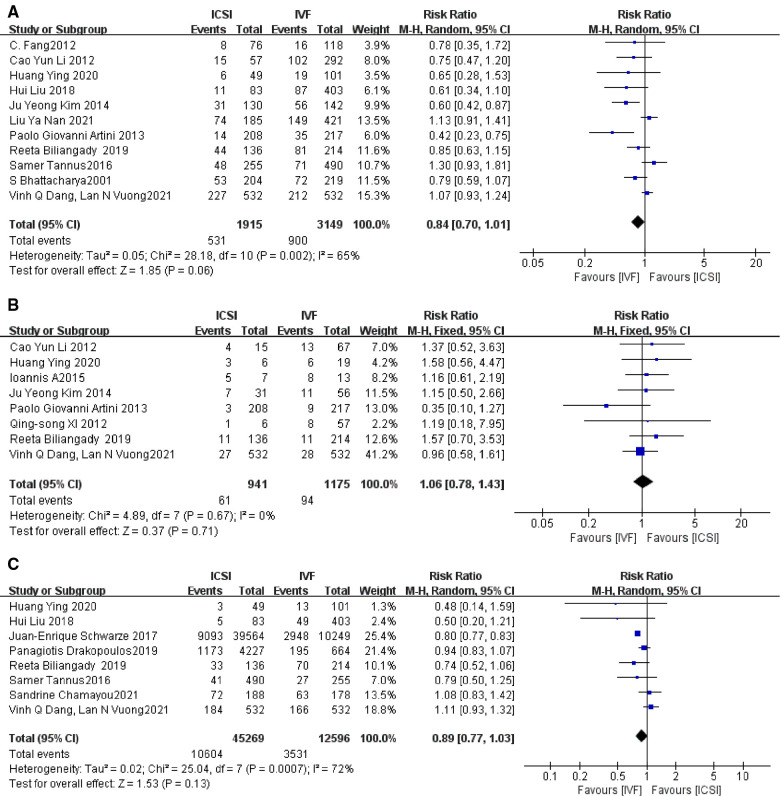
(**A**) Clinical pregnancy rate; (**B**) miscarriage rate; (**C**) live birth rate of fresh embryo transfer.

There was significant heterogeneity in the clinical pregnancy rate of fresh embryo implantation in 11 studies (4, 5, 9, 10, 13, 16, 20, 22, 23, 25, 27), when excluding the study with the greatest weight (27) there was no difference in the results. *I*^2^ = 65%, RR = 0.84, 95% CI [0.70, 1.01], *P* = 0.06 (Figure 4A).

##### Subgroup analysis of clinical pregnancy rate

Subgroup analysis was performed according to patients from different countries, with Chinese as a group and foreigners as a group, as shown in the Supplementary Figure S5, the heterogeneity was moderate in the Chinese group and greater in the foreigners, probably due to the difference in the types of studies, with the Chinese group being a retrospective cohort study, while in the foreigner's group, the study by S Bhattacharya and Vinh Q Dang was a randomized controlled study, and the rest of the studies were retrospective cohort studies; it is also possible that the reason for this is the difference in findings. However, the *P*-values were >0.05, indicating that the clinical pregnancy rates of fresh embryos after ICSI and IVF in patients with non-male factor infertility were similar in different countries.

##### Miscarriage rates

The presence of no heterogeneity among the eight studies (5, 9, 11, 13, 20–22, 27), when the study with the greatest weight (27) was excluded, the results showed no difference with an RR value of 1.06, 95% CI [0.78, 1.43], *P* = 0.71 > *P* = 0.05, Figure 4B, indicating that the miscarriage rates in the two groups were not statistically significant, i.e., the ICSI group miscarriage rate and that of the IVF group were not different.

##### Subgroup analysis of miscarriage rates

Subgroup analysis was performed according to the different countries of each study, divided into Chinese and foreigner groups, and the results are shown in Supplementary Figure S6: The heterogeneity between the two groups at subgroup analysis was almost 0 and the *P*-values were all greater than 0.05, indicating that there was no statistically significant difference in the abortion rates compared between the two subgroups.

##### Live birth rate of fresh embryo transfer

The heterogeneity among the eight studies (9, 10, 12, 22–24, 27, 30) was more skewed and the results did not differ when the study with the greatest weight (12) was excluded. The *I*^2^, RR = 0.89, 95% CI [0.77, 1.03], *P* = 0.13 > *P* = 0.05, Figure 4C, indicating that there was no statistically significant difference between the ICSI group and the IVF group in the comparison of the live birth rate of fresh embryo transfer.

##### Subgroup analysis of live birth rate of fresh embryo transfer

Subgroup analysis was performed according to mean age, with a group ≤35 years old and a group >35 years old. In the Panagiotis study, fresh embryos and cumulative live birth rates were analyzed according to different ovarian response categories and different mean ages, so the subgroups were grouped according to different ages. The results are shown in Supplementary Figure S7. In the group with mean age ≤35 years, *I*^2^ = 41, the heterogeneity between studies was moderate, RR = 1.00, 95% CI [0.89, 1.11], *P* = 0.95 > *P* = 0.05, indicating that the live birth rate of fresh embryo transfer in the ICSI group was not statistically significant compared to the IVF group in the group with a younger mean age. In the group with mean age >35 years, the *I*^2^ value was 39% with moderate heterogeneity between the two studies, RR = 0.80, 95% CI [0.78, 0.83], *P* < 0.00001, indicating that the live birth rate of fresh embryo transfer in ICSI versus IVF was statistically significant in the older group, and the RR value was 0.80 < 1, indicating that the live birth rate of fresh embryo transfer in IVF was superior to the ICSI group. In several studies of older patients with non-male factor infertility, some studies showed higher live birth rates with IVF than with ICSI, although not statistically significant, all showed higher live birth rates with IVF than with ICSI.

#### A meta-analysis of neonatal outcomes

##### Preterm delivery rate

As shown in Supplementary Figure S8, *I*^2^ = 0, There was no heterogeneity between the four studies (8, 16, 27, 29), and the results did not differ after excluding the study with the greatest weight (27), RR = 0.92, 95% CI [0.67, 1.26], *P* = 0.61 > *P* = 0.05, There was no statistical significance between the ICSI and IVF groups.

##### Low neonatal weight rate

As shown in Supplementary Figure S9, *I*^2^ = 30%, heterogeneity was low among the four studies (8, 16, 27, 29), fixed effects model was used and the results did not differ after excluding the study with the greatest weight (8), RR value was 1.13, 95% CI was [0.80, 1.61], *P* = 0.48 > *P* = 0.05, ICSI group, and IVF group was not statistically significant.

##### Sensitivity analysis and publication bias

Sensitivity analyses were performed by excluding the study with the greatest weight in each outcome, and the excluded outcomes were stable as previously described, except for the total fertilization rate, where the reasons for this difference could be that Juan-Enrique Schwarze performed for 49,813 ART cycles, performed ICSI for 39,564 cycles, and performed IVF for 10,249 cycles, without randomised allocation, and that ICSI was performed only in couples with the poorest prognosis or who experienced a history of IVF failure, which could have contributed to the bias. Finally for studies with less heterogeneity funnel plots can be constructed, with symmetrical funnel plots indicating no significant publication bias and symmetrical funnel plots indicating no significant publication bias. When running Rev Man 5.4 software, the funnel plots for complete fertilization failure rate, neonatal preterm birth rate and neonatal low birth weight rate were symmetrical, as shown in Supplementary Figures S10–S1S2, with no significant publication bias.

## Discussion

### Summary of main results

The results of our meta-analysis of forest plots showed no differences in fertilization rate, total fertilization failure rate, good quality embryo rate, fresh embryo implantation clinical pregnancy rate, fresh embryo transfer live birth rate, miscarriage rate, neonatal preterm birth rate, and neonatal low birth weight rate in those treated with ICSI compared to IVF. However, the implantation rate of IVF was superior to that of ICSI, and in the subgroup analysis of the live birth rate of fresh embryo transfer, those older than 35 years of age who underwent IVF had a higher live birth rate.

The reasons for the debate between ICSI and conventional IVF are as follows: it has been argued that the advantages of ICSI over conventional IVF are that it may facilitate the selection of sperm with good morphological characteristics in terms of sperm selection (13) and that the operation accurately bypasses the zona pellucida and increases the fertilization of fertilized eggs (31) avoids fertilization failure rates (32), increases the quality of potentially usable embryos (1) and is considered to be the first choice in ICSI for couples with unexplained infertility (33) and poor ovarian response (34). However, the fact that ICSI was not found to be superior to IVF. Fertilization is a complex multi-step and multifactorial process. Failure of fertilization can occur at any step of the process such as sperm zona pellucida binding, gamete fusion, oocyte activation, and sperm depolymerization, and IVF is the closest form of fertilization to the natural union (35).

In case of poor ovarian response, the selection of more mature MII stage oocytes for ICSI may improve the fertilization rate due to poor egg quality; however, the possible mechanism by which ICSI is not superior to IVF in terms of fertilization rate is that ICSI causes mechanical damage to the oocyte, leading to fertilization failure. In contrast, conventional IVF assays for oocyte maturation at 16–18 h after fertilization, where the oocyte-ovarian complex remains intact in culture, allow more oocytes to mature and fertilize (23). The reason why fertilization rates did not improve in the ICSI group of older women in our meta-analysis may be that most of the included studies were retrospective, and patients with more complex causes of infertility or the worst prognosis received ICSI, thus highlighting the advantages of ICSI, and many clinical studies have concluded that fertilization rates are higher with ICSI than with IVF (4, 6, 14), so the exploration of fertilization rates requires continuous clinical research; the higher implantation rate of IVF than ICSI may also be related to the natural selection of sperm that does not damage the oocyte (21). In studies on clinical pregnancy rate, miscarriage rate, and live birth rate, neither IVF nor ICSI had any effect on the developmental potential of fertilized eggs, and the intrinsic developmental potential of oocytes determines the embryonic developmental potential (10), i.e., although oocytes of poor quality can be fertilized normally by ICSI, they still do not progress toward high-quality embryos and live births.

Therefore, ICSI is not superior to IVF in terms of clinical pregnancy rate, miscarriage rate, and live birth rate. The neonatal outcomes of ICSI and IVF are similar, and the number of people receiving ICSI and IVF in the randomized controlled study of VQ was both 532, and the proportion of low neonatal weight rate and preterm birth rate were both similar, which indicates that ICSI and IVF have less impact on neonates. Regarding the safety discussion of ICSI, the sperm being injected is chosen arbitrarily, the operation of ICSI ignores the zona pellucida binding and oocyte fusion steps completely (36), and suboptimal sperm may lead to the transmission of undesirable genetic traits, resulting in genomic or phenotypic abnormalities in the offspring (37). Embryologists have a subjective view of sperm phenotypic traits and do not know the genetic quality of the sperm (38), which may increase the probability of embryonic aneuploidy occurrence if the sperm is of poor genetic quality or if exogenous materials such as bacteria and viruses adhering to the sperm surface are injected into the oocyte cytoplasm together with the sperm during the injection. Therefore, more clinical trials are needed to explore the safety of ICSI.

ICSI has been suggested as a strategy to manage low oocyte yield in cases of inadequate ovarian reserve (POR), reduced ovarian reserve (DOR), and male-free infertility (39). 10 studies were included by the authors, but there were inconsistencies in outcomes such as fertilization rates and clinical pregnancy rates between IVF and ICSI. Three studies chose the gold standard of ART, cumulative live birth rate, for comparison, with only one study showing a superior result for IVF over ICSI (10), one finding no differenc (30) and one finding that cumulative live birth rate was independent of the fertilization method (37). Some studies have shown that fertilization rates are low in patients with endometriosis because their extracted oocytes may not mature easily *in vitro*, exhibiting altered morphology and low cytoplasmic mitochondrial content (37). ICSI is therefore recommended, but studies have also found little difference in outcome between the use of IVF and ICSI (14). Some studies have found that autoimmune disorders also affect couples of reproductive age, such as the formation of anti-sperm antibodies detected in women with unexplained infertility (40). Which interfere with sperm penetration of the zona pellucida and cause IVF failure (39), and as research continues to be uncovered, it has been found that ICSI may overcome the problems posed by ASA (41).

Compared with other studies, the results of our meta-analysis, similar to the outcome of the randomized controlled study by Haas (42), showed no significant differences between ICSI and IVF in terms of fertilization rates and good quality embryo rates. A meta-analysis comparing pregnancy rates in elderly patients with non-male factor infertility (43) showed that ICSI was not superior to IVF in women older than 38 years of age in terms of fertilization rates. Ting showed no difference between ICSI and IVF in terms of clinical pregnancy rates, implantation rates, and live birth rates (44). But our meta-analysis differed significantly between ICSI and IVF in terms of implantation rate and live birth rate in non-male factor infertile patients older than 35 years. Kylie concluded that fertilization rates, clinical pregnancy rates, and live birth rates were higher with IVF than with the ICSI group in patients with non-male factor infertility (45). More clinical trials are needed to demonstrate the differences between ICSI and IVF.

### Limitations of this study

The limitation of this study is that only fresh embryos were selected for the comparison of ICSI and IVF, the outcome indicators of frozen embryos were not studied, the outcome indicators are still lacking and the study findings need to be improved. In addition, fewer randomized controlled trials and more retrospective cohort studies were included in this study, and the methods of randomization, concealment, and blinding of some studies have not been clarified, which may lead to greater heterogeneity. The source of bias may also be the uneven quality of the included literature. Therefore, a large number of randomized controlled studies are still needed to confirm the outcome of the studies.

## Conclusion

The present meta-analysis focused on the embryo laboratory outcome, pregnancy outcome, and neonatal outcome of ICSI versus IVF in patients with non-male factor infertility, followed by exploring the effectiveness and safety of the application of ICSI. Compared with IVF, those who received ICSI treatment had a higher fertilization rate, complete fertilization failure rate, good-good quality embryo rate, fresh embryo implantation clinical pregnancy rate, miscarriage rate, fresh embryo transfers live birth rate, and preterm neonatal birth rate, and low neonatal weight rate were not different. However, the implantation rate of IVF was better than that of ICSI, and in the subgroup analysis of the live birth rate of fresh embryo transfer, there was a higher live birth rate of IVF in those older than 35 years. The advantages of this study are that the included studies are from the last 20 years, with a large period and large total sample size to ensure the credibility of the meta-study results, and the inclusion of clinical comparisons of perinatal and neonatal outcomes in patients with non-male factors, and a more in-depth study of ICSI.

## Data Availability

The original contributions presented in the study are included in the article/**Supplementary Material**, further inquiries can be directed to the corresponding author/s.

## Supplementary material

SUPPLEMENTARY FIGURE S1

Subgroup analysis of fertilization rate.

SUPPLEMENTARY FIGURE S2

Total fertilization failure rate Removal of maximum weight.

SUPPLEMENTARY FIGURE S3

Subgroup analysis of total fertilization failure rate.

SUPPLEMENTARY FIGURE S4

Subgroup analysis of good-good quality embryo rate.

SUPPLEMENTARY FIGURE S5

Subgroup analysis of clinical pregnancy rate.

SUPPLEMENTARY FIGURE S6

Subgroup analysis of miscarriage rate.

SUPPLEMENTARY FIGURE S7

Subgroup analysis of the live birth rate of fresh embryo transfer.

SUPPLEMENTARY FIGURE S8

Preterm delivery rate.

SUPPLEMENTARY FIGURE S9

Low birth weight rate.

SUPPLEMENTARY FIGURE S10

Funnel figure of Total fertilization failure rate.

SUPPLEMENTARY FIGURE S11

Funnel figure of Preterm delivery rate.

SUPPLEMENTARY FIGURE S12

Funnel figure of Low neonatal weight rate.
