# Supplementary figures and images for: Impact of intracytoplasmic sperm injection in women with non-male factor infertility: A systematic review and meta-analysis
Source: Front Reprod Health. 2022 Oct 28;4:1029381. doi: 10.3389/frph.2022.1029381 (PMC9650435; doi:10.3389/frph.2022.1029381)

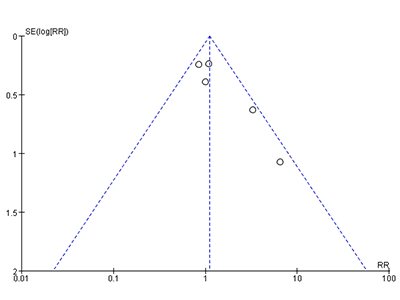

Supplement: Supplementary file 1 [file Datasheet1.zip › Supplementary Figure 10.tif]

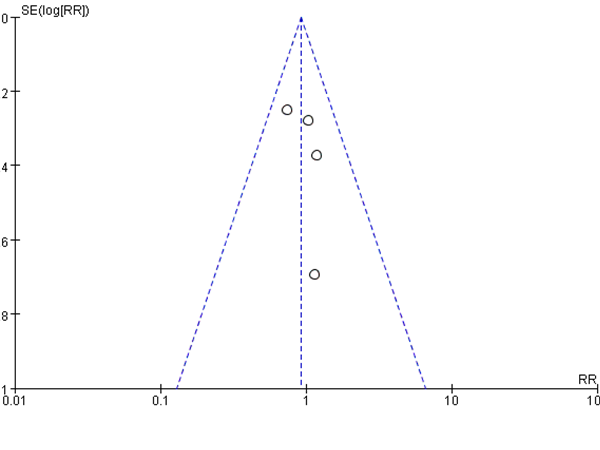

Supplement: Supplementary file 1 [file Datasheet1.zip › Supplementary Figure 11.tif]

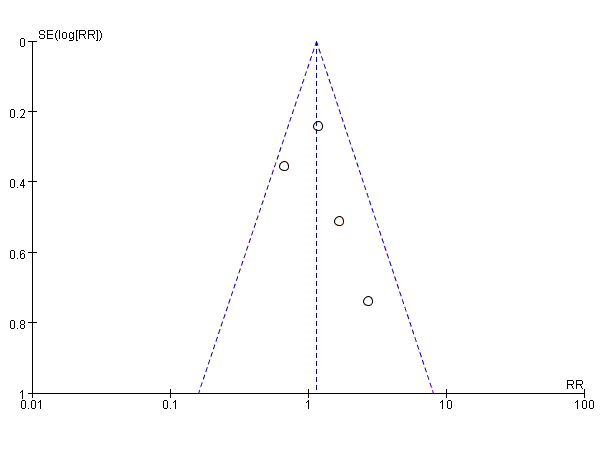

Supplement: Supplementary file 1 [file Datasheet1.zip › Supplementary Figure 12.tif]
